# Effect of a single pill concept on clinical and pharmacoeconomic outcomes in cardiovascular diseases

**DOI:** 10.1093/ehjcvp/pvae059

**Published:** 2024-07-31

**Authors:** Burkhard Weisser, Sven Wassmann, Hans-Georg Predel, Roland E Schmieder, Anton Gillessen, Thomas Wilke, Jörg Blettenberg, Olaf Randerath, Antje Mevius, Michael Böhm

**Affiliations:** Institute of Sports Science, Christian-Albrechts-University of Kiel, Kiel 24098, Germany; Cardiology Pasing, Munich, Germany; Faculty of Medicine, University of the Saarland, Homburg, Saar, Germany; Institute of Cardiology and Sports Medicine, German Sport University, Cologne 50933, Germany; Department of Nephrology and Hypertension, University Hospital Erlangen, Friedrich Alexander University Erlangen Nürnberg, Erlangen 91054, Germany; Department of Internal Medicine, Herz-Jesu-Hospital, Münster 48149, Germany; Institute for Pharmacoeconomics and Pharmaceutical Logistics (IPAM)/Institute for Pharmacoeconomics and Pharmaceutical Logistics, Wismar 23966, Germany; Practice Dr J. Blettenberg, Lindlar 51789, Germany; Medical Department, Apontis Pharma Deutschland GmbH & Co. KG, Monheim 40789, Germany; Institute for Pharmacoeconomics and Pharmaceutical Logistics (IPAM)/Institute for Pharmacoeconomics and Pharmaceutical Logistics, Wismar 23966, Germany; Clinic for Internal Medicine III, University Clinic of Saarland, Saarland University, Homburg, Saar 66421, Germany

**Keywords:** Arterial hypertension, Dyslipidemia, Cardiovascular disorders, Adherence, Single pill concept, Cardiovascular outcomes

## Abstract

**Aims:**

Our study aimed to assess whether a single pill concept (SPC) is superior to a multi-pill concept (MPC) in reducing cardiovascular (CV) events, all-cause death, and costs in CV patients.

**Method and results:**

Anonymized medical claims data covering 2012–2018, including patients with hypertension, dyslipidaemia, and CV diseases who started a drug therapy either as SPC or identical MPC were analysed after 1:1-propensity score matching. Hospitalizations with predefined CV events, all-cause mortality, and costs were studied in 25 311 patients with SPC and 25 311 patients with MPC using incidence rate ratios (IRRs) and non-parametric tests for continuous variables.

IRRs were significantly lower for SPC: stroke (IRR = 0.77; 95% CI 0.67–0.88; *P* < 0.001), transitory ischaemic attack (IRR = 0.61; 95% CI 0.48–0.78; *P* < 0.001), myocardial infarction (IRR = 0.76; 95% CI 0.63–0.90; *P* = 0.0016), coronary artery disease (IRR = 0.66; 95% CI 0.57–0.77; *P* < 0.001), heart failure (IRR = 0.59; 95% CI 0.54–0.64; *P* < 0.001), acute renal failure (IRR = 0.54; 95% CI 0.56–0.64; *P* < 0.001), all cause hospitalization (IRR = 0.72; 95% CI 0.71–0.74; *P* < 0.001), CV hospitalization (IRR = 0.63; 95% CI 0.57–0.69; *P* < 0.001), and all-cause mortality (IRR = 0.62; 95% CI 0.57–0.68; *P* < 0.001). Mean time to first events and time to death were also in favour of SPC. Mean total costs were 4708€ for SPC vs. 5.669€ for MPC, respectively (mean ratio 0.830, *P* < 0.001).

**Conclusion:**

SPC is associated with lower incidence rates of CV events, time to CV events, and all-cause death, and is superior regarding pharmacoeconomic parameters and should therefore become standard of care to improve outcomes and reduce healthcare costs.

## Graphical Abstract

**Graphical Abstract:**
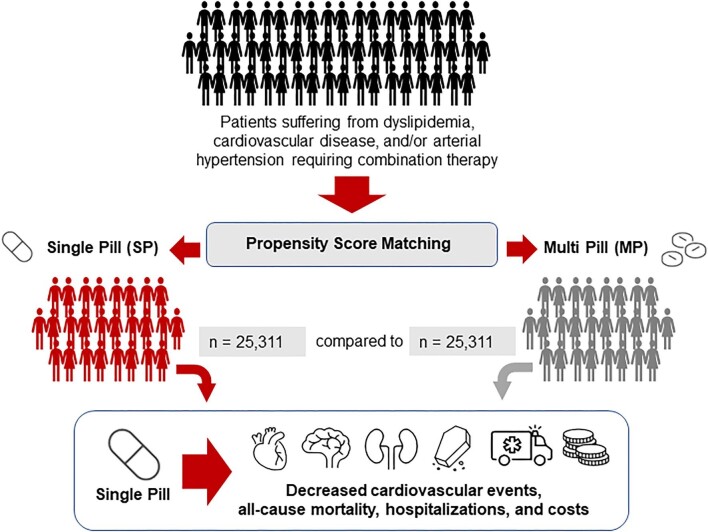

## Introduction

Hypertension, dyslipidaemia, and diabetes are the most prevalent risk factors leading to cardiovascular (CV) morbidity and mortality.^1–6^ The risk of stroke, myocardial infarction (MI), heart failure, and chronic kidney disease (CKD) has been shown to be reduced by control of high blood pressure with antihypertensive medications and lipid-lowering drugs.^7,8^ Patients at high CV risk usually require a combination of different drugs to reach their treatment targets.^9–12^ For each of these drugs, their clinical benefit was demonstrated in clinical trials. However, despite protective effects on CV outcomes, pharmacological treatment is often suboptimal.^1,2,4,5^ One explanation is a lack of adherence as a very common phenomenon in primary care,^10–15^ which strongly influences blood pressure and lipid control, with a relevant impact on the CV risk imposing a large financial burden on health care systems.^16–18^ Current guidelines for the management of arterial hypertension or secondary CV prevention recommend combination drug treatments with single pills (SP).^19,20^ This concept is expected to improve adherence to treatment and, as a consequence, to reduce the risk of adverse CV outcomes associated with these clinical conditions which was shown for patients suffering from hypertension.^21^ Aim of our study was to assess whether a SP concept (SPC) is clinically and pharmacoeconomically superior to a multi-pill concept (MPC) with identical drugs in reducing CV events and all-cause mortality in a large real-world population.

## Methods

We analysed anonymized medical claims data from patients aged 18 years or older with CV disease or high risk insured by AOK PLUS, a German statutory health fund, and treated with a combination as SPC or identical MPC during the time period 1 July 2012 to 30 June 2018. The dataset provided information on socio-demographic characteristics of patients, inpatient and outpatient care as well as all documented diagnoses, prescriptions of medications, and other data such as prescriptions of outpatient aids and devices.

Patients were included in the analysis if they were continuously insured (July 2012 to June 2018, death as only exception from this rule) and had at least one inpatient or two outpatient claims (in two different quarters) of at least one of the following diseases in 1 July 2012 to 30 June 2017: hypertension, coronary heart disease (CHD), hyperlipidaemia, MI, HF, stroke, transient ischaemic attack, or peripheral artery disease. The International Code of Diseases (ICD) was chosen for disease classification because the validity of recording and coding is high in statutory health fund data, especially for inpatient and prescription data, as they are directly relevant for reimbursement of hospitals/pharmacies, and regularly checked by external agencies. We predefined the following combinations which are commonly used to treat the predefined diseases and were available as SPC or identical MPC in this population: bisoprolol/amlodipine, valsartan/amlodipine, candesartan/amlodipine, valsartan/amlodipine/hydrochlorothiazide, ramipril/amlodipine, ezetimibe/atorvastatin, acetylsalicylic acid (ASA)/atorvastatin/ramipril. Patients who started their combination therapy between 1 July 2013 and 30 June 2017 as MPC or SPC were included in the analysis. Observation of patients started at the date of the first prescription of combination therapy (index date), either as SPC or MPC. Index date for the SPC group was the first prescription of a respective SPC. An MPC was assumed to have been prescribed if there were claims of all agents of the targeted combination therapy within 90 days; index date was the prescription date of the last (second/third) agent in that combination.

A propensity score matching (PSM) was done to account for baseline differences. In the PSM analysis, patients in the SPC cohort were, per subgroup, separately matched to patients in the MPC cohort. Propensity scores were calculated using logistic regression (group affiliation as dependent variable) including age, gender, and Charlson Comorbidity Index (CCI) without age factor as fixed independent variables. Covariates in the PSM regarding medication were a number of prescribed medications (counting each agent—ATC code level 5—with at least two prescriptions), use of antihypertensive agents, oral antidiabetic agents, insulin, antiarrhythmic agents, non-steroidal anti-inflammatory drugs, antiplatelets, lipid lowering agents, drugs for peptic ulcer and GORD, cardiac glycosides, oral corticosteroids, and benzodiazepine derivatives. All were based on information related to the index date or a 12 month baseline period. Furthermore, 28 different variables that are plausible as predictors of CV outcomes available in the database describing the CV event risk or the general comorbidity profile of the above patients, were included as independent variables. A backward elimination approach was used to eliminate any variables that did not reach significance in explaining group exposition (*P* > 0.1); in such cases, these variables were excluded from the specific PSM models. As each covariate included in the PSM analysis was expected to affect both treatment assignment and the outcomes of interest, PSM quality was assessed in two ways: (1) standardized differences between comparison groups were used to assess the balance of covariates after matching (number of variables with—still—significant differences between SPC and MPC patients); (2) the incidence of specific events that were expected to be independent of the drug treatment received, such as later knee/hip replacement in the follow-up period, were compared.

Patients were followed up from the index date until one of the following events, whatever came first: end of data availability (30 June 2018), all-cause death, therapy discontinuation defined as a gap in drug supply of at least 60 days, based on the defined daily dose per agent, in case of an MPC a gap of 60 days for at least one of the combination agents led to censoring in that respect, switch from SPC to MPC or vice versa.

The following acute and non-planned clinical outcomes reported as main diagnoses were captured: hospitalization with stroke, transitory ischaemic attack, MI, coronary artery disease, heart failure, acute and/or chronic renal failure, all cause hospitalization, CV hospitalization, and all-cause mortality.

Based on a patient-specific follow-up period since index date, we counted the number of events per observed 100 patient years and, based on this, calculated incidence rate ratios (IRRs) for the exposure of a patient to the SPC vs. MPC cohorts. In addition, we calculated unadjusted hazard ratios (HRs) based on unadjusted Cox regression models. Third, the percentage of event-free patients over time with regard to the outcomes of interest as well as with regard to a composite outcome consisting of all cause hospitalizations was depicted in Kaplan Meier curves using log rank tests for testing statistical significance of differences between the observed cohorts.

To address the issue of confounding, two additional analyses were conducted: an analysis of the number of events in a Poisson regression and a multivariable Cox regression analysis both based on the unmatched SPC/MPC samples within above cohorts. Results were reported as coefficients for SPC vs. MPC (Poisson regression) and adjusted HRs (aHRs, Cox regression). All variables included in the PSM procedure were included in these models as independent variables using a backward elimination approach.

All reported *P*-values were two-sided, and 95% CIs were calculated for IRRs, HRs, Poisson-coefficients and aHRs by applying independent *t*-tests or Wilcoxon Rank Sum tests, where applicable. For categorical variables, the Chi Squared Test was performed. All descriptive analyses were performed with Microsoft SQL Server 2014 and Microsoft Excel 2016. All other statistical analyses were performed with STATA/MP 13.1 and SPSS 17.0.

## Regulatory aspects

As the study addressed an anonymized dataset, no ethical review and no informed consent of patients were needed. However, the study protocol was reviewed by the scientific steering committee (Institut für Pharmakoökonomie und Arzneimittellogistik (IPAM)/Institute for Pharmacoeconomics and Pharmaceutical Logistics, Wismar; AOK PLUS—die Gesundheitskasse für Sachsen und Thüringen; GB Arzneimittel/Heilmittel, Dresden; Institute of Sports Science, Christian-Albrechts-University of Kiel; Cardiology Pasing, Munich; Institute of Cardiology and Sports Medicine, German Sport University, Cologne; Department of Nephrology and Hypertension, University Hospital Erlangen, Friedrich Alexander University Erlangen Nürnberg; Department of Internal Medicine, Herz-Jesu-Hospital, Münster; Practice Dr J. Blettenberg, Lindlar; Clinic for Internal Medicine III, University Clinic of Saarland, Saarland University, Homburg/Saar).

## Results

**Figure 1:**
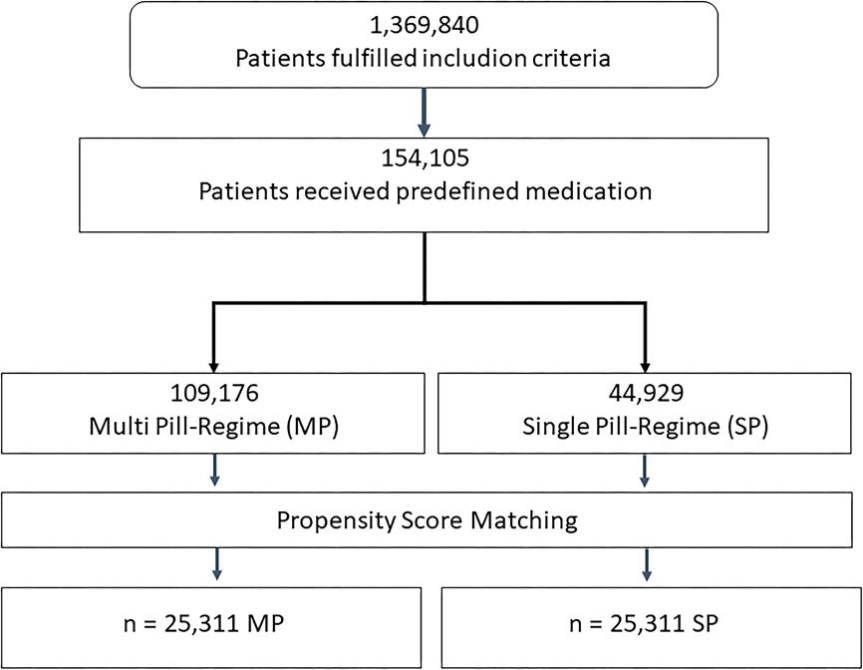
Patient flow. Figure 1 highlights the consort flow diagram.

**Figure 2:**
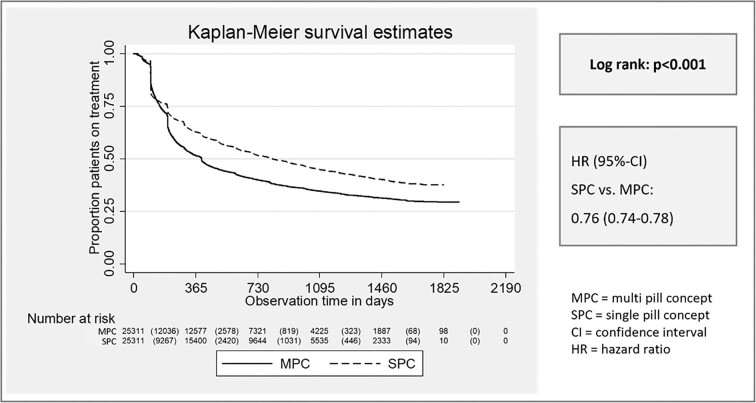
Time to non-persistence. Figure 2 shows the proportion of patients persistent to medication over the observational period. Comparisons are done between matched SPC vs. MPC cohorts.

A total number of 50 622 patients (25 311 patients on SPC vs. 25 311 patients on MPC) aged ≥18 years treated with SPC or MPC with identical drugs were followed up for at least 1 year or until death (*Figure 1*). Inclusion diagnoses of the matched cohorts are provided in Supplementary material online, *Table S1*, and baseline characteristics of the matched cohorts are given in Supplementary material online, *Table S2*. No significant differences in baseline characteristics were observed after PSM. The proportion of patients persistent to treatment one year after initiation of treatment was 63.4% under SPC and 52.4% under MPC, respectively (*P* < 0.001) and was consistently higher under SPC during the whole observational period [HR (95%–CI) SPC vs. MPC 0.76 (0.74–0.78), Log Rank: *P* < 0.001, *Figure 2*].

**Figure 3:**
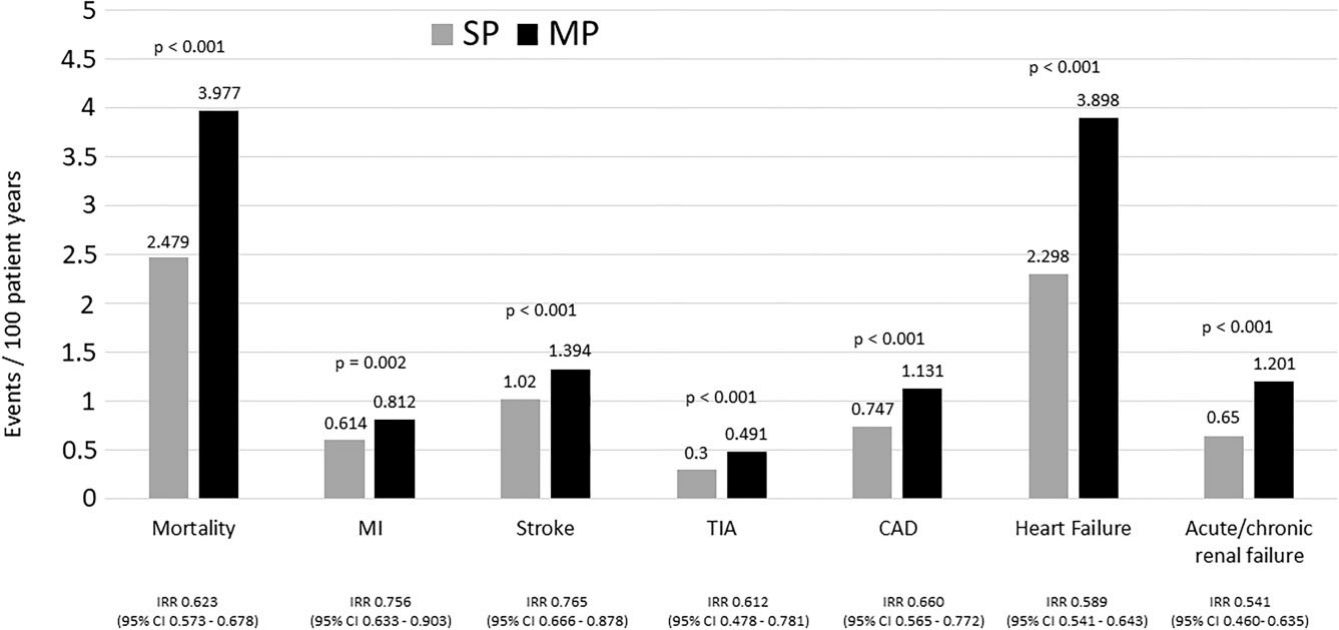
Event rates per treatment group. Figure 3 shows the number of all-cause mortality, myocardial infarction, stroke, transitory ischaemic attack, coronary artery disease, heart failure, acute/chronic renal failure per observed 100 patient years in the respective cohorts. Comparisons are done between matched SPC vs. MPC cohorts.

**Figure 4:**
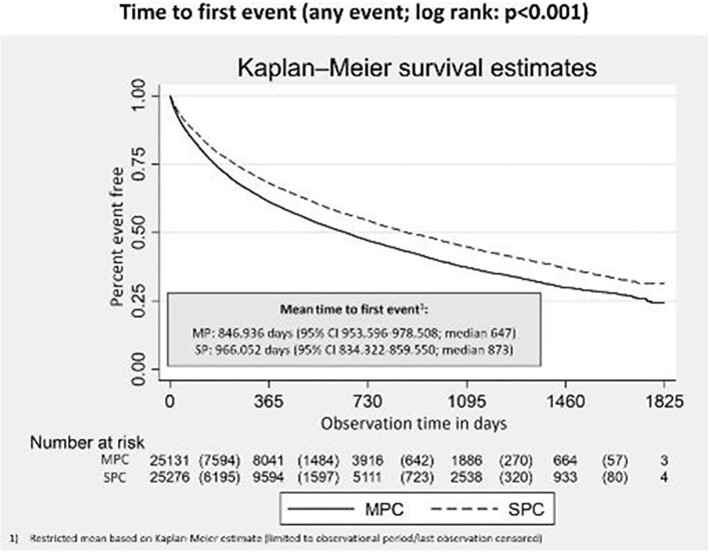
Time to first Event. Figure 4 shows the proportion of patients that are event free over the observational period. Events that were observed were hospitalizations with diagnosis of myocardial infarction, stroke, transitory ischaemic attack, coronary artery disease, heart failure, acute and chronic renal failure, any cardiovascular hospitalization, all cause hospitalization, all-cause mortality. Comparisons are done between matched SPC vs. MPC cohorts.

**Figure 5:**
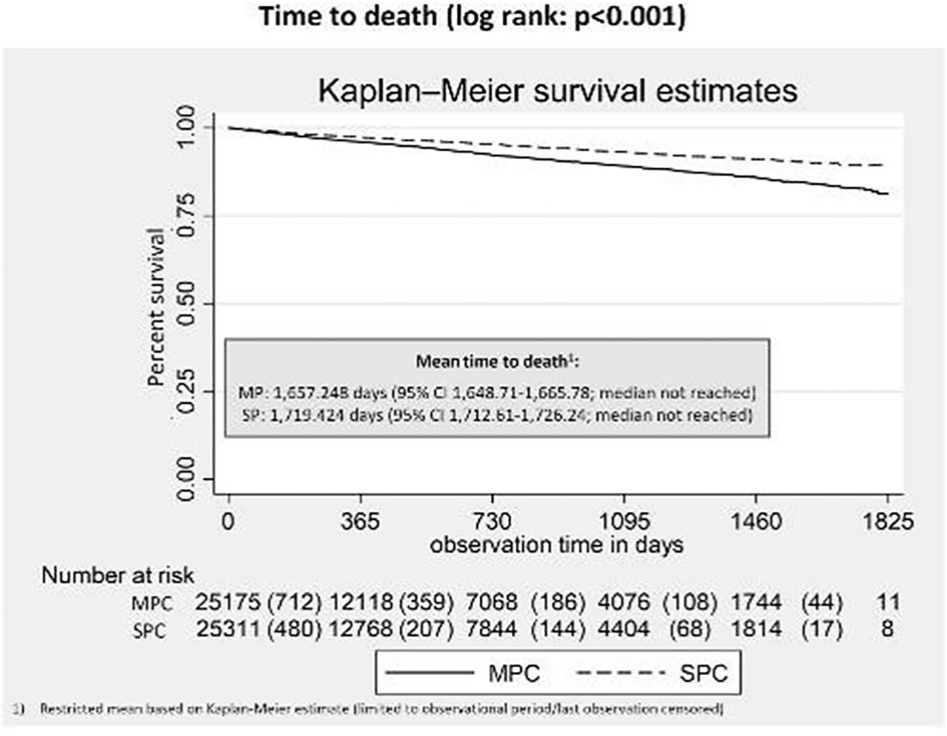
Time to death. Figure 5 shows the proportion of patients surviving over the observational period. Comparisons are done between matched SPC vs. MPC cohorts. Patients were followed up from index date until one of the following events, whatever came first: End of data availability (30 July 2018), all-cause death, therapy discontinuation defined as gap in drug supply of at least 60 days, based on the defined daily dose (DDD) per agent, in case of a MPC a gap of 60 days for at least one of the combination agents led to censoring in that respect, switch from SPC to MPC or vice versa.

Comparisons were done on nine CV outcomes. In all comparisons, significantly lower IRR were identified for SPC, confirmed by comparison of Kaplan-Meier estimates: hospitalizations with stroke (IRR = 0.77; 95% CI 0.67–0.88; *P* < 0.001), hospitalizations with transitory ischaemic attack (IRR = 0.61; 95% CI 0.48–0.78; *P* < 0.001), hospitalizations with MI (IRR = 0.76; 95% CI 0.63–0.90; *P* = 0.0016), hospitalizations with coronary artery disease (IRR = 0.66; 95% CI 0.57–0.77; *P* < 0.001), hospitalizations with heart failure (IRR = 0.59; 95% CI 0.54–0.64; *P* < 0.001), hospitalizations with acute renal failure (IRR = 0.54; 95% CI 0.56–0.64; *P* < 0.001), all cause hospitalization (IRR = 0.72; 95% CI 0.71–0.74; *P* < 0.001), CV hospitalization (IRR = 0.63; 95% CI 0.57–0.69; *P* < 0.001), and all-cause mortality (IRR = 0.62; 95% CI 0.57–0.68; *P* < 0.001) (*Figure 3*). The mean time to first events (*Figure 4*) and time to death (*Figure 5*) were also in favour of SPC (any event: SPC 966.052 days/median 873; MPC 846.936 days/median 647; death: SPC 1719.424 days; MPC 1657.248 days; log rank for both comparisons: *P* < 0.001).

Health care utilization for CV causes was lower on SPC as judged by outpatient visits [SP 3.96 vs. MP 4.02, IRR 0.985, *P* < 0.001], specialist visits (SPC 0.30 vs. MPC 0.36, IRR 0.829, *P* > 0.1), days in hospital (SPC 0.54 vs. 0.82 MPC, IRR 0.659, *P* < 0.001), prescriptions (SPC 2.24 vs. MPC 2.79, IRR 0.804, *P* < 0.001), and days absent from work (SPC 2.8 vs. MPC 3.34, IRR 0.837, *P* < 0.001). Similar results were observed for general medical health care utilization: outpatient visits (SPC 4.42 vs. MPC 4.55, IRR 0.971, *P* < 0.001), specialist visits (SPC 0.45 vs. MPC 0.53, IRR 0.854, *P* = 0.002), days in hospital (SPC 3.77 vs. MPC 5.19, IRR 0.728, *P* < 0.001), number of prescriptions (SPC 5.79 vs. MPC 6.72, IRR 0.862, *P* < 0.001), and days absent from work (SPC 7.84 vs. MPC 7.78, IRR 1.007, *P* = 0.007). The comparison for the medication costs was divided into costs for the focus medication (according to the cohort definitions) and costs for other CV-related medication. The mean costs for any outpatient medication ranged between 1140€ and 2653€ (SPC) and between 1138€ and 2701€ (MPC). The mean costs were slightly higher for the SPC groups (IRR 1.028) but not statistically significant. Mean costs related to focus diagnosis per patient per year were 1214€ in the SPC vs. 1225€ in the MPC [mean ratio (MR) 0.991, *P* = 0.085], mean total costs were 4708€ under SPC vs. 5.669€ under MPC, respectively (MR 0.830, *P* < 0.001).

## Discussion

The major finding of this study in 50 622 patients was that the concept of an SP combination use in CV disease reduced total mortality and improved incidence rates and time to event of nine clinical outcomes significantly compared to an MPC with identical substances. Patients treated with SPC were more and longer persistent to medication than with MPC, which might explain the better prognosis under SPC. Health care utilization and related costs were also in favour of SPC.

In the past, improvement in adherence to medication has been observed for the use of SPC.^1,6,7,15–17^ In a meta-analysis comparing adherence to treatment under an SP compared with the identical MPC in patients with CV disease, SPC showed higher adherence and persistence to medication after 6 months, 12 months, and 18 months.^22^ However, data on outcomes are sparse.

A previous study in patients with CV diseases comparing SPC with MPC demonstrated a reduction in CV events in favour of SPC.^23^ An analysis with renin-angiotensin system inhibitor combinations given as a single pill or multiple pills confirmed this observation in patients with hypertension.^21^ In the retrospective, observational NEPTUNO study using data from electronic-health records, patients were distributed into four different cohorts: a single pill containing ASA/atorvastatin/ramipril (case cohort), identical mono components taken separately. A total of 6456 patients (1614 patients per cohort) were analysed. After 2 years, the risk of recurrent MACE was lower in the single pill cohort compared to all control groups with better blood pressure and LDL-cholesterol control.^24^ These studies were hampered by the use of differently potent drugs between SPC and MPC. The recently published SECURE study in patients after MI within the previous 6 months randomized to a single pill containing ASA/atorvastatin/ramipril or usual care. A total of 2499 patients were enrolled and followed for a median of 36 months. In the single pill group, patients had a significantly lower risk of major adverse CV events and CV deaths than patients under usual care.^25^

Our study extends those findings of previous studies.^26,27^ It shows that risk reduction linked to specific substances is superior when used in a single pill, providing a concept in CV disease treatment in general, which should be used when patients need combination therapy.

The observed reduction of health care utilization and costs in favour of SPC compared to MPC further supports this concept. Less CV events require less cost and time-intensive interventions. Borghi et al.^28^ designed a microsimulation to project health outcomes between 2020 and 2030 for populations with hypertension managed according to current treatment practices (CTP), single drug treatment with dosage titration and sequential addition of other agents. In this model, simulated outcomes of mortality, CKD, stroke, ischaemic heart disease, and disability-adjusted life years were estimated for 1 000 000 patients for each of the four treatment pathways.^28^ SPC improved clinical outcomes over the other treatment regimens.^28^ Ten-year projections indicated SPC is showing the greatest overall benefits, which is linked to improved adherence to medication.^28^

The SP concept will also have an influence on pill burden, which was also identified as a risk factor for the development of CV events. Derington and colleagues investigated the association of baseline medication burden and clinical outcomes in a subanalysis of the SPRINT trial. High medication burden was associated with worse intensive systolic blood pressure control and higher rates of CV disease events and serious adverse events.^29^

Our study has some limitations. Differences between the compared SPC and MPC groups could be a potential limitation as it could reflect differences in intensity of health care in individual patients. However, we quality-checked our PSM by comparing hip and knee replacement surgery frequencies as a potential independent non-CV outcome. We could not detect any significant differences between the two groups. Second, a higher number of patients in the initial dataset got a prescription for MPC compared to SPC. The exclusion of many MPC patients from the analyses could lead to a bias. However, we addressed this by two additional sensitivity analyses based on unmatched cohorts, with consistent results. Third, we did not have access to clinical data like blood pressure values so that we used claims-based proxies to identify the outcomes of interest. To address potential weaknesses of the study resulting from this, we observed multiple outcomes attributed to CV complications associated with the underlying diseases. Another limitation is that due to non-availability of data, we could not explore the pill burden of target patients outside of the CV medications. So, our analysis shows the relative effect of reducing a CV-related pill burden, independent of the baseline pill burden of a patient. Further studies should explore whether this baseline pill burden is an effect modifier regarding SPC. Limiting is also, that disease severity was not accounted for in the propensity matching of patients due to data unavailability. However, we tried to minimize a disease-related bias by including the additional covariates in the PSM regarding comorbidities which were CHA2DS2-VASc, top five comorbidities that are not covered by the CCI/CHA2DS2-VASc, level of care, number of hospitalizations with any CV diagnosis as main diagnosis, number of hospitalizations with any non-CV diagnosis as main diagnosis, participation in a disease management program, confirmed diagnosis of dementia, confirmed diagnosis of affective disorders, confirmed mental, and behavioural disorders due to psychoactive substance/alcohol use.

Finally, as is the case for all retrospective database analyses, diagnoses, or outcome misclassification, although non-differential, constitutes an additional limitation. To minimize the risk resulting from this limitation and in line with previous similar studies, we only captured confirmed events requiring an acute hospital admission and a documentation of the event itself as the main diagnosis.

## Conclusion

Compared to a MPC with identical substances, SPC is associated with a lower incidence of CV events and lower all-cause mortality, time to CV events and death as well as with lower health care utilization and costs. Therefore, SPC should become standard of care in the treatment of hypertension, dyslipidaemia, and secondary prevention of CV disease whenever available.

## Supplementary Material

pvae059_Supplemental_Files

## Notes

**Conflicts of interest:** MB reports support from Astra-Zeneca, Bayer, Boehringer-Ingelheim, Medtronic, Novartis, ReCor, Servier and Vifor. RES received grants to the Institution from Amgen, Ablative Solution, AstraZeneca Boehringer Ingelheim, IPPmed, Medtronic, Novartis, NovoNordisk, Recor, and he is on the speaker and advisor bureau of Ablative Solutions, Apontis, AstraZeneca, Bayer, Boehringer Ingelheim, Lilly, Medtronic, Merck, NovoNordisk, Novartis, Recor, Servier. TW did studies for various pharmaceutical companies and received grants from Cytel Inc., a consultancy working for these companies. SW is on the speaker and advisory bureau of Amgen, Apontis, AstraZeneca, Bayer, Berlin Chemie, Bristol-Myers Squibb, Boehringer Ingelheim, Daiichi Sankyo, Lilly, Novartis, Pfizer, and Vifor. OR is member of the Medical Department of APONTIS PHARMA Deutschland GmbH & Co. KG, Germany.

## Data availability

Because of the sensitive nature of the data collected for this study, requests to access the dataset from qualified researchers trained in human subject confidentiality protocols may be sent to the Institut für Pharmakoökonomie und Arzneimittellogistik e.V., Alter Holzhafen 19, 23 966 Wismar/Germany.
