# Supplementary figures and images for: Effect of a single pill concept on clinical and pharmacoeconomic outcomes in cardiovascular diseases
Source: Eur Heart J Cardiovasc Pharmacother. 2024 Jul 31;10(8):686–93. doi: 10.1093/ehjcvp/pvae059 (PMC11724138; doi:10.1093/ehjcvp/pvae059)

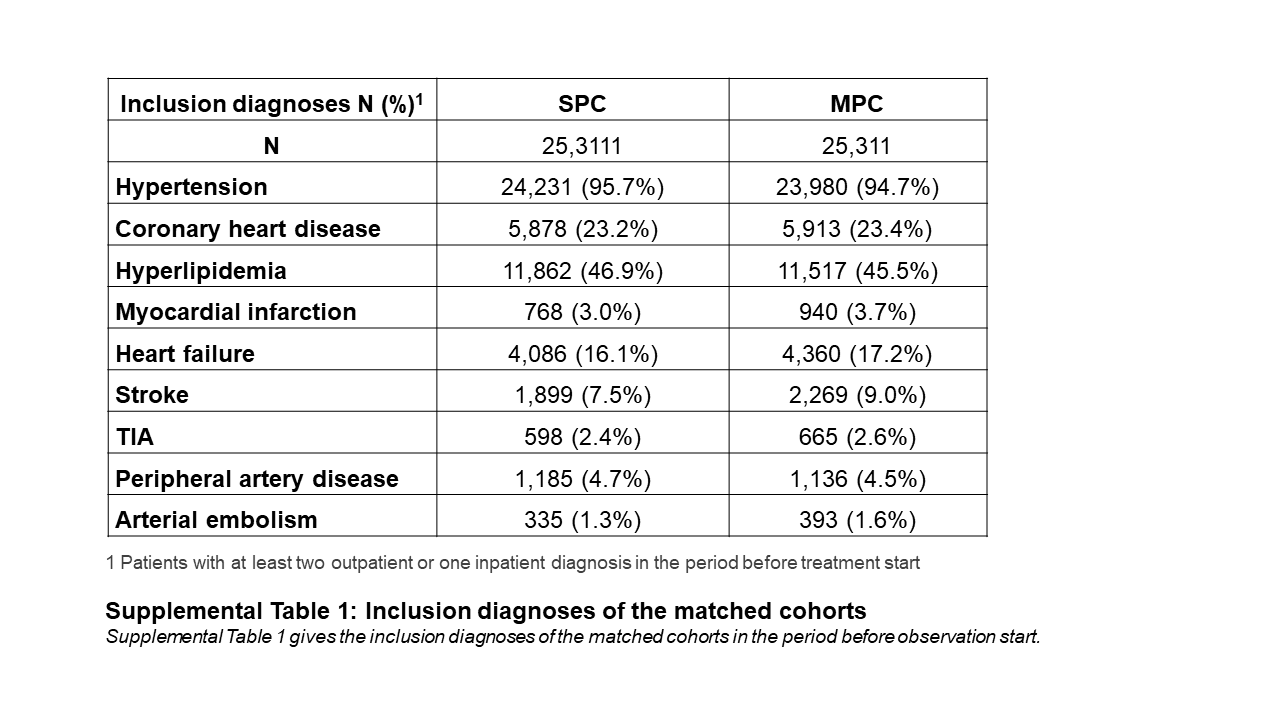

Supplement: pvae059_Supplemental_Files [file pvae059_supplemental_files.zip › Supplemental Table 1.tif]

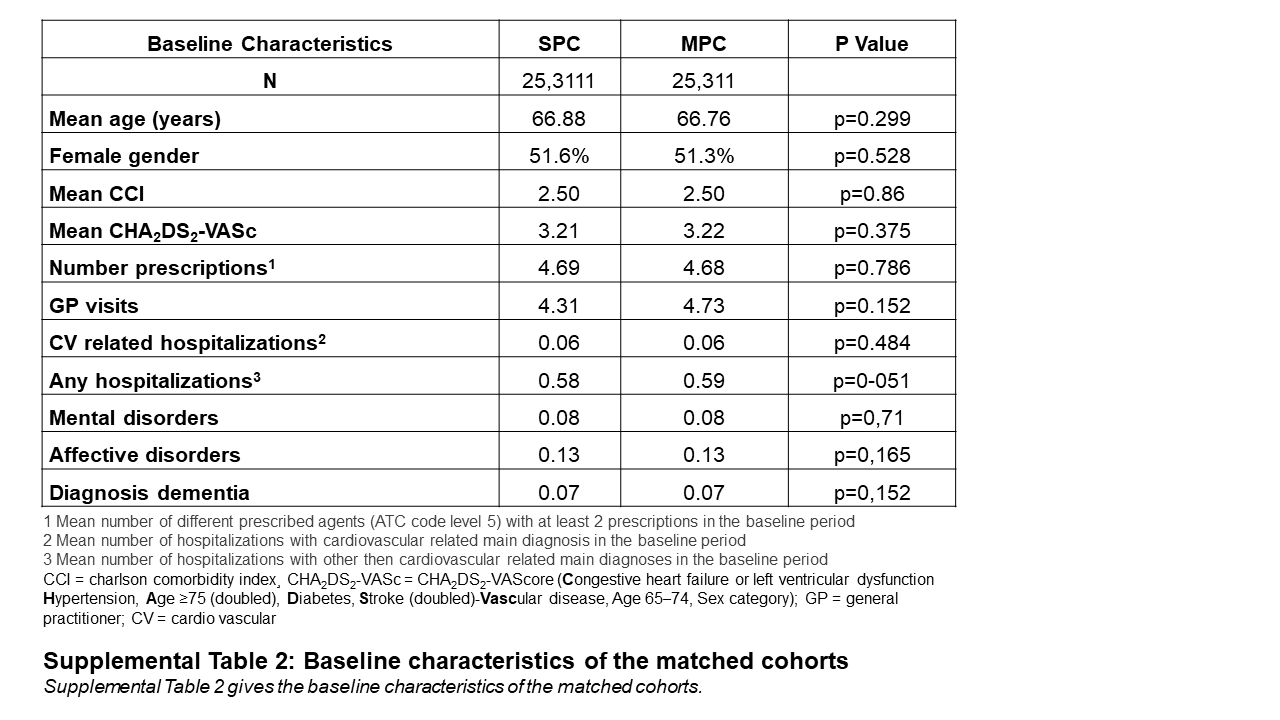

Supplement: pvae059_Supplemental_Files [file pvae059_supplemental_files.zip › Supplemental Table 2.tif]
